# Dietary Fat Influences the Expression of Contractile and Metabolic Genes in Rat Skeletal Muscle

**DOI:** 10.1371/journal.pone.0080152

**Published:** 2013-11-11

**Authors:** Wataru Mizunoya, Yohei Iwamoto, Bungo Shirouchi, Masao Sato, Yusuke Komiya, Farzaneh Rahimi Razin, Ryuichi Tatsumi, Yusuke Sato, Mako Nakamura, Yoshihide Ikeuchi

**Affiliations:** 1 Department of Bioresource Sciences, Graduate School of Agriculture, Kyushu University, Fukuoka, Japan; 2 Department of Bioscience and Biotechnology, Graduate School of Agriculture, Kyushu University, Fukuoka, Japan; University of Minnesota Medical School, United States of America

## Abstract

Dietary fat plays a major role in obesity, lipid metabolism, and cardiovascular diseases. To determine whether the intake of different types of dietary fats affect the muscle fiber types that govern the metabolic and contractile properties of the skeletal muscle, we fed male Wistar rats with a 15% fat diet derived from different fat sources. Diets composed of soybean oil (n-6 polyunsaturated fatty acids (PUFA)-rich), fish oil (n-3 PUFA-rich), or lard (low in PUFAs) were administered to the rats for 4 weeks. Myosin heavy chain (MyHC) isoforms were used as biomarkers to delineate the skeletal muscle fiber types. Compared with soybean oil intake, fish oil intake showed significantly lower levels of the fast-type MyHC2B and higher levels of the intermediate-type MyHC2X composition in the extensor digitorum longus (EDL) muscle, which is a fast-type dominant muscle. Concomitantly, MyHC2X mRNA levels in fish oil-fed rats were significantly higher than those observed in the soybean oil-fed rats. The MyHC isoform composition in the lard-fed rats was an intermediate between that of the fish oil and soybean oil-fed rats. Mitochondrial uncoupling protein 3, pyruvate dehydrogenase kinase 4, and porin mRNA showed significantly upregulated levels in the EDL of fish oil-fed rats compared to those observed in soybean oil-fed and lard-fed rats, implying an activation of oxidative metabolism. In contrast, no changes in the composition of MyHC isoforms was observed in the soleus muscle, which is a slow-type dominant muscle. Fatty acid composition in the serum and the muscle was significantly influenced by the type of dietary fat consumed. In conclusion, dietary fat affects the expression of genes related to the contractile and metabolic properties in the fast-type dominant skeletal muscle, where the activation of oxidative metabolism is more pronounced after fish oil intake than that after soybean oil intake.

## Introduction

Skeletal muscle is the largest organ in the human body and comprises approximately 40% of body weight. In the skeletal muscle of most animals, there are two major fiber types: type 1 fibers (slow-twitch oxidative, red muscle) and type 2 fibers (fast-twitch glycolytic, white muscle). Type 1 fibers are rich in mitochondria, possess high oxidative capacity, and are resistant to fatigue. Muscles enriched in type 1 fibers, such as the soleus, perform sustained and tonic contractile activities, like postural tension. Conversely, type 2 muscle fibers exhibit high glycolytic metabolism and fatigue easily. Muscles enriched in type 2 fibers, such as the extensor digitorum longus (EDL), are typically involved in intense and rapid activities of short duration. Fiber type composition varies greatly between individuals, resulting in differences in exercise performance, fatigue resistance, and metabolic capacity in humans [1]. Studies in animal models have also demonstrated a strong relationship between muscle fiber type and the development of diabetes and obesity [2,3].

Skeletal muscle fiber types are generally classified according to myosin heavy chain (MyHC) isoforms. In adult rodent skeletal muscles, four MyHC isoforms have been identified: MyHC1, 2A, 2X, and 2B [4]. MyHC1 is expressed in type 1 muscle fibers. Type 2 fibers are further subdivided into type 2A, 2X, and 2B muscle fibers, in which MyHC2A, 2X, and 2B are preferentially expressed, respectively. Type 2A and 2X fibers have intermediate characteristics between type 1 and type 2B fibers. Although type 2X fibers are sometimes defined as fast-twitch glycolytic fibers, type 2B fibers have an even stronger fast-twitch glycolytic phenotype than type 2X fibers [5–7].

The muscle fiber type in adult muscles can switch in response to hormonal status (particularly that of thyroid hormones) [8] or activation/contraction patterns, e.g., external electrical stimulation [9], paralysis due to spinal cord injury [10], denervation [11], cross-reinnervation (fast muscle reinnervated by a slow nerve and vice versa) [12], mechanical unloading [13], and food components [14]. Endurance training is also a well-known stimulus that induces fast-to-slow fiber type transition [15].

The fatty acid profiles of fat derived from plants, animals, and fish are unique. The dietary fats of plant origin are rich in either n-6 polyunsaturated fatty acids (PUFA) (soybean oil) or n-3 PUFA (flaxseed oil), of animal origin are rich in saturated fatty acids (lard), and of fish origin are rich in n-3 PUFA (fish oil). To date, different types of dietary fat has been shown to alter numerous processes involved in the regulation of energy metabolism. Fish oil containing high n-3 PUFA, including eicosapentaenoic acid (EPA) and docosahexaenoic acid (DHA) have been reported to improve lipid metabolism. In humans, the oxidation of lipids is increased by the consumption of dietary fish oil [16], which suggests that n-3 PUFA can reduce adiposity. The anti-obesity effect of n-3 PUFA reflects the metabolic changes in several tissues, such as stimulation of lipid oxidation and inhibition of lipogenesis in the liver [17,18], stimulation of fatty acid oxidation in the muscle [19], and adaptive thermogenesis mediated by mitochondrial uncoupling protein 1 (UCP1) in brown fat [20,21]. Dietary vegetable oils enriched in n-6 PUFA, such as linoleic acid are reported to lower serum cholesterol levels [22], although the increases in the ratio of n-6/n-3 PUFA, characteristic of the Western diet, could potentiate inflammatory processes and consequently predispose to or exacerbate many inflammatory diseases [23].

Since the composition of the muscle fiber types closely reflects its metabolic property, it is important to determine whether the muscle fiber types are affected by different types of dietary fat. If this were indeed the case, a diet composition that enhances aerobic metabolism would result in higher numbers of slow or intermediate-type fibers. In this study, we examined whether different dietary fat sources—soybean oil (n-6 PUFA-rich), fish oil (n-3 PUFA-rich), or lard (low in PUFAs)—could affect the composition and metabolism-related genes in skeletal muscle fibers in both slow-type dominant (soleus) and fast-type dominant (EDL) muscle tissues. In addition, we measured the expression levels of transcription factors that regulate the muscle fiber types in these muscle tissues.

## Materials and Methods

### Animals and diets

Male Wistar rats (7 weeks old) weighing 210–230 g were purchased from a commercial supplier (Kyudo, Tosu, Japan). The rats were individually housed in stainless-steel wire-mesh cages in an animal room at 22 ± 2°C at 50 ± 10% humidity under an artificial lighting system of 12-h light and 12-h darkness (lights on from 0800 to 2000) and were fed a commercial diet (Type CRF-1 of Oriental Yeast Co., Tokyo, Japan) ad libitum for 1 week to acclimatize them to the environment. Following the acclimatization period, rats were divided into three groups (soybean oil-, fish oil-, and lard-fed groups, n = 6 each) so that each respective group had the same initial body weight and average food intake during the acclimatization period. All diets were prepared based on a modified AIN-93G composition containing 15% (w/w) soybean oil, lard, or fish oil; the detailed diet compositions are shown in Table 1. The diets were prepared as pellets by Oriental Yeast Co. Ltd., Japan, then sealed, light-shielded, and stored at 4°C until use. Table 2 shows the fatty acid compositions of the soybean oil, lard, and fish oil. Each rat was given a fresh diet every day for 4 weeks by pair-feeding. Food intake and body weight were measured daily. The animals were allowed free access to water during the entire experimental period. All experimental procedures were performed according to the Guidelines for Animal Experiments for the Faculty of Agriculture and the Graduate Course of Kyushu University and the law [No.105] and notification [No.1] of the Japanese Government, and with the ethical approval of Animal Care and Use Committee, Kyushu University (protocol 05-002-01).

**Table 1:** The composition of experimental diets (g/kg).

Ingredient	Soybean oil diet	Fish oil diet	Lard diet
α-Corn starch	387.2	387.2	387.2
Sucrose	120.5	120.5	120.5
Casein	241.0	241.0	241.0
Soybean oil	150.0	-	-
Fish oil	-	150.0	-
Lard	-	-	150.0
Cellulose powder	50.0	50.0	50.0
Mineral mix (AIN-93G-MX)	35.0	35.0	35.0
Vitamin mix (AIN-93-VX)	10.0	10.0	10.0
L-Cystine	3.6	3.6	3.6
Choline bitartrate	2.5	2.5	2.5
tert-Butylhydroquinone	0.14	0.14	0.14

**Table 2:** The fatty acid composition of the dietary fats in experimental diets (%).

	Fatty acid	Soybean oil	Fish oil	Lard
12:0	lauric acid	-	0.07	2.83
14:0	myristic acid	5.55	8.13	2.58
15:0	pentadecylic acid	-	2.20	-
16:0	palmitic acid	5.66	15.94	25.07
16:1	palmitoleic acid	-	9.89	2.38
17:0	margaric acid	-	2.51	-
18:0	stearic acid	10.61	3.01	7.31
18:1	oleic acid	20.66	11.47	46.42
18:2n-6	linoleic acid	50.42	0.86	10.06
18:3n-3	α-linolenic acid	5.18	0.57	0.71
20:1	gadoleic acid	-	0.71	-
20:4n-6	arachidonic acid	-	0.96	0.38
20:5n-3	eicosapentaenoic acid	-	20.18	-
22:5n-3	docosapentaenoic acid	-	2.17	-
22:6n-3	docosahexaenoic acid	-	8.11	-
	other fatty acids	1.92	13.21	2.26
	SFA	21.8	31.9	37.8
	MUFA	20.7	22.1	48.8
	PUFA	55.6	32.9	11.2
	n–6/n-3	9.7	0.1	14.8

SFA; saturated fatty acid, MUFA; monounsaturated fatty acid, PUFA; polyunsaturated fatty acid

### Tissue and blood sampling

After the treatment period, the rats were anesthetized via intraperitoneal injection of pentobarbital sodium (50 mg/kg body weight) and sacrificed by decapitation. Blood was rapidly collected from the neck. Sera were collected from the blood and kept at -80°C until use for assays. Serum concentrations of glucose (Glucose-CRII, Wako Pure Chemical Industries, Osaka, Japan), free fatty acids (FFA) (NEFA C Test Wako, Wako Pure Chemical Industries), triglycerides (Triglyceride E Test Wako, Wako Pure Chemical Industries), and ketone bodies (Ketone Test A and B, Sanwa Chemical Institute, Nagoya, Japan) were measured using commercial kits. The EDL, soleus, tibialis anterior, plantaris, and gastrocnemius muscles; epididymal, perirenal, and mesenteric fat; brown adipose tissue; and liver, heart, and kidneys were removed and weighed. The EDL and soleus muscles were rapidly frozen in liquid nitrogen and stored at -80°C until analysis. 

### Preparation of protein and RNA samples

EDL and soleus muscles were ground to a powder with a mortar and pestle cooled with liquid nitrogen. For the protein assay, weighed, frozen, powdered muscles (approx. 50 mg) were homogenized with a motor-driven small pestle in an SDS solution containing 10% SDS, 40 mM DTT, 5 mM EDTA, and 0.1 M Tris-HCl buffer (pH 8.0), in which Protease Inhibitor Cocktail for Use with Mammalian Cell and Tissue Extracts (Nacalai Tesque, Inc., Kyoto, Japan) was added at 1:100. The sample homogenates were heated in boiling water for 3 min. Total protein concentrations were assayed using BCA Protein Assay Reagent (Pierce), with bovine serum albumin as a standard. The samples were diluted in 2 × sample buffer (100 mM DTT, 4.0% SDS, 0.16 M Tris-HCl (pH 6.8), 43% glycerol, and 0.2% bromophenol blue) and dH_2_O to give final protein concentrations of 10 ng/µL (myosin heavy chain isoform assay) or 8 µg/µL (Western blotting) in 1 × sample buffer. For the mRNA assay, total RNA was isolated from powdered EDL and soleus muscles (approx. 50 mg) using TRIzol Reagent (Invitrogen) according to the manufacturer's protocol. Briefly, muscle tissue was homogenized in 1 mL TRIzol Reagent using a Polytron homogenizer. Cooled chloroform was added and separated by centrifugation. The aqueous phase containing RNA was transferred to a clean tube, and the RNA was precipitated by adding isopropanol and incubating at room temperature for 10 min. After another centrifugation, the RNA pellet was washed with 75% ethanol and finally dissolved in 20 µL of diethylpyrocarbonate-treated water. The RNA concentration was determined spectrophotometrically, and 0.6 µg of total RNA was reverse transcribed by a reverse-transcriptase SuperScript III (Invitrogen) using Oligo d(T)16 primer (Applied Biosystems, Carlsbad, CA, USA). The protein and reverse-transcribed cDNA samples were stored at -80°C until use for assays.

### Analysis of MyHC isoform composition

EDL and soleus protein samples were subjected to high-resolution SDS-polyacrylamide gel electrophoresis for the assessment of MyHC isoform content as described in detail by Mizunoya et al. [24]. Briefly, the gel was composed of 8% acrylamide (the acrylamide/Bis ratio was 99:1) and contained 35% (v/v) glycerol. After samples (50 ng) were loaded, electrophoresis was performed at a constant voltage of 140 V for 22 h at 4°C. Gels were stained with Silver Stain Kanto III (Kanto Chemical Co. Inc., Tokyo, Japan) and dried. The bands were captured on an image scanner, and the relative content of MyHC isoforms was quantified by densitometry using ImageJ 1.34s software (Rasband W, National Institutes of Health, USA). MyHC isoforms were identified according to their different migration rates (MyHC1 > 2B > 2X > 2A).

### Real-time quantitative RT-PCR

Real-time quantitative PCR using Roche LightCycler1.5 was performed under the TaqMan probe detection format and standardized according to β-actin expression. All primers and appropriate probes were designed and chosen by ProbeFinder (version 2.35 for rat, Roche) with an intron-spanning assay, with the exception of mitochondrially encoded cytochrome c oxidase 2 (MTCO2) and myogenic differentiation 1 (MyoD). The primer sets and the Universal Probe Library TaqMan probes (Roche) for these genes are listed in Table 3. Threshold cycles (Ct) were determined as the PCR cycle at which an increase in fluorescence above a baseline signal was first detected. The annealing temperature was set to 60°C in all cases. Transcript levels of genes related to (i) energy metabolism (myoglobin, lipoprotein lipase (LPL), UCP3, and pyruvate dehydrogenase kinase 4 (PDK4)); (ii) mitochondrial proteins (porin, MTCO2, UCP3, and PDK4); and (iii) transcription factors (peroxisome proliferator-activated receptor δ (PPARδ), forkhead box O1 (FOXO1), MyoD, and myogenin), and a coactivator (PGC1α) for muscle fiber type regulation were analyzed. Genes were analyzed by using a standard curve constructed from a serial dilution of cDNA aliquots pooled from one randomly chosen sample. To clarify differences in the four MyHC isoform amounts within the same muscle, the MyHC isoforms were analyzed using the ΔCt method [25] to compare the expression relative to β-actin expression. For other genes, values were standardized to the β-actin expression level and expressed as the fold-change in gene expression relative to the soybean oil-fed group.

**Table 3:** Primer/probe sets used for real-time quantitative PCR.

mRNA		Sequence (5'-3')	Probe #^1^	Amplicon (nt)	Access no.
MyHC1	Forward	GAGGAGAGGGCGGACATT	95	88	NM_017240
	Reverse	ACTCTTCATTCAGGCCCTTG			
MyHC2A	Forward	AAATCTTACAAGAGACAAGCTGAGG	73	67	NM_001135157
	Reverse	TGCGGAACTTGGATAGATTTG			
MyHC2X	Forward	CCTGCAGCTCCAAGTTCAGT	112	69	NM_001135158
	Reverse	ATCAGCTGGTCGCATCTTTC			
MyHC2B	Forward	TGCCTCCTTCTTCATCTGGT	46	111	NM_019325
	Reverse	AGCAGCCTCCCCAAAAAC			
myoglobin	Forward	GGGACAACATGCTGCTGAG	110	113	NM_021588
	Reverse	TCTTCAGGACTTGGATGATGAC			
LPL	Forward	CAGAGAAGGGGCTTGGAGA	58	85	NM_012598
	Reverse	TTCAGCAGGGAGTCAATGAA			
UCP3	Forward	CCCCTACACTGTATGCTGAGG	79	77	NM_013167
	Reverse	AGAAAGGAGGGCATGAATCC			
PDK4	Forward	GAGCTGTTCTCCCGCTACAG	120	84	NM_053551
	Reverse	AGTTCTCTCACAGGCATTTTCTG			
porin	Forward	TTTTCGGCCAAAGTGAACA	120	118	NM_031353
	Reverse	CACCCGCATTGACGTTCT			
MTCO2	Forward	GACGCCCAAGAAGTAGAAACAA	14	72^2^	N/A
	Reverse	GGAGGGAAGGGCAATTAGAA			
PPARδ	Forward	TCGAGTTTGCTGTCAAGTTCA	50	127	NM_013141
	Reverse	TGGATGGCTTCCACCTGT			
PGC1α	Forward	GAAGCGGGAGTCTGAAAGG	29	76	NM_031347
	Reverse	GTAAATCACACGGCGCTCTT			
FOXO1	Forward	TCAGGCTAGGAGTTAGTGAGCA	68	95	NM_001191846
	Reverse	GGGGTGAAGGGCATCTTT			
MyoD	Forward	CCCTGTTGTTTGTGGAGACA	50	69^2^	NM_176079
	Reverse	CTGTGGGAAAGAGTGGGTGT			
myogenin	Forward	CCTTGCTCAGCTCCCTCA	63	94	NM_017115
	Reverse	TGGGAGTTGCATTCACTGG			
β-actin	Forward	CTGGCTCCTAGCACCATGA	63	76	NM_031144
	Reverse	TAGAGCCACCAATCCACACA			

1 Universal Probe Library probe number (Roche Applied Science)

2 Not intron-spanning

MyHC, myosin heavy chain; LPL, lipoprotein lipase; UCP3, uncoupling protein 3; PDK4, pyruvate dehydrogenase kinase 4; MTCO2, mitochondrially encoded cytochrome c oxidase 2; PGC1α, peroxisome proliferator-activated receptor gamma coactivator 1α; FOXO1, forkhead box O1; PPARδ, peroxisome proliferator-activated receptor δ; MyoD, myogenic differentiation 1.

### Western blot analysis

For the Western blot analysis, EDL and soleus proteins (40 µg) were loaded onto 12% polyacrylamide gels, subjected to electrophoresis, and transferred to Hybond ECL nitrocellulose membranes (GE Healthcare) for 4 h at 38 V (constant voltage). The membranes were blocked with 5% skim milk diluted in Tris-buffered saline containing 0.1% Tween 20 (TBS-T) for 45 min at room temperature. The blots were then incubated overnight at 4°C with gentle agitation in dilutions of primary antibodies. The following antibodies were used: mouse monoclonal anti-myoglobin (Sigma M7773, 1:1000); anti-pan-actin (Chemicon MAB1501 (clone C4), 1:3000000); anti-porin (Abcam ab14734, 1:4000000); rabbit polyclonal anti-UCP3 (Abcam ab3477, 1:20000); anti-PGC1α (Calbiochem 516557, 1:10000); and rabbit monoclonal anti-FOXO1 (Epitomics 1874-1, 1:2000). The primary antibodies were diluted in Can Get Signal solution 1 (Toyobo). After washing three times in TBS-T for 10 min, the membranes were incubated for 1 h with horseradish peroxidase-conjugated rat anti-mouse IgG (Jackson ImmunoResearch 287695) or swine anti-rabbit IgG (Dako P0399) at a 1:5000 dilution with Can Get Signal solution 2 (Toyobo) and then washed three times in TBS-T for 10 min. The blots were developed via enhanced chemiluminescence (ECL Western Blotting Reagents, RPN2106, GE Healthcare) and Hyperfilm ECL (GE Healthcare). The optical densities of the bands were normalized to actin as a loading control.

### Analysis of fatty acid composition in serum and muscle

Lipids in the serum and gastrocnemius muscle were extracted by the method described by Folch et al. [26]. Gastrocnemius muscle was used because it represents a fast-dominant muscle that is made of mainly fast-type fibers and has sufficient mass for the experimental analysis. Methyl esterification of the extracted lipids was performed using a boron trifluoride methanol solution. The methyl-esterified fatty acid was subjected to a gas chromatography-flame ionization detector (GC-FID) system (GC-17, Shimadzu, Tokyo, Japan) equipped with a capillary column (Omegawax320, 30 m × 0.25 mm ID, Sigma-Aldrich Japan, Tokyo, Japan) and a Chromatopac integrator (C-R6A; Shimadzu) to analyze the fatty acid composition and relative ratios. The injection port and detector temperatures were 250°C. The column temperature was 200°C. Helium was used as the carrier gas at a flow rate of 65 cm/s. The fatty acid species was identified by comparison with the retention time of a fatty acid methyl ester (FAME) standard solution (Supelco 37 Component FAME Mix, Sigma-Aldrich Japan) and a single FAME, C20:3n-9 (Cayman Chemical, MI, USA). The relative content of the respective fatty acids was calculated from the GC-FID chromatogram.

### Statistical analysis

Data are expressed as the mean ± SE. Comparisons among the three experimental groups were performed by one-way ANOVA followed by a Tukey–Kramer multiple-comparison test, when appropriate. A value of p < 0.05 was considered significant. Statistics were calculated with Excel-Toukei 2006 (Social Survey Research Information Co., Ltd., Tokyo, Japan).

## Results

### Body and organ weights

Growth parameters, such as body weight, food intake amount, skeletal muscle weights, and organ weights, for each experimental diet group are shown in Table 4. The final body weight and body weight gain were similar among the three groups. There was no significant difference in body weight throughout the four-week experimental period. However, food efficiency in the fish oil-fed rats was significantly higher than that in the lard-fed rats. Several skeletal muscle weights did not show any significant difference among the three diet groups, but mesenteric fat, liver, and kidney weights were significantly higher in the fish oil-fed rats than in the lard-fed rats. In soybean oil-fed rats, the levels of these parameters were intermediate of those of fish oil-fed and lard-fed rats.

**Table 4:** Growth performance and organ weights in rats fed each diet (g).

	Soybean oil			Fish oil			Lard		
Initial body weight	286.8	±	4.2	286.5	±	3.4	286.0	±	3.1
Final body weight	462.7	±	12.5	475.8	±	15.1	448.7	±	12.0
Body weight gain	175.9	±	8.6	189.2	±	12.7	162.7	±	10.1
Total food intake	684.9	±	21.7	708.9	±	31.4	695.1	±	27.6
Food efficiency†	0.26	±	0.01^ab^	0.27	±	0.01^a^	0.23	±	0.01^b^
EDL	0.37	±	0.01	0.36	±	0.01	0.37	±	0.01
Soleus	0.30	±	0.02	0.31	±	0.01	0.30	±	0.01
Gastrocnemius	4.31	±	0.13	4.25	±	0.17	4.27	±	0.09
Plantaris	0.89	±	0.05	0.88	±	0.03	0.87	±	0.03
TA	1.42	±	0.04	1.42	±	0.05	1.42	±	0.02
Epididymal fat	9.21	±	0.28	7.81	±	0.59	8.81	±	0.82
Perirenal fat	14.59	±	1.34	11.96	±	1.23	14.73	±	1.40
Mesenteric fat	9.03	±	0.74^ab^	10.97	±	0.61^a^	8.29	±	0.76^b^
Brown adipose tissue	1.02	±	0.05	1.08	±	0.05	0.88	±	0.07
Liver	19.26	±	1.25^ab^	21.62	±	1.33^a^	17.11	±	0.70^b^
Kidneys	3.13	±	0.08^ab^	3.46	±	0.11^a^	2.83	±	0.14^b^
Heart	1.25	±	0.03	1.34	±	0.03	1.41	±	0.13

Values are means ± SE for 6 rats. Different superscripts indicate there is a significant difference between two groups (p < 0.05).

EDL; extensor digitorum longus, TA; tibialis anterior.

† food efficiency = body weight gain (g) / total food intake (g)

### Serum energy metabolites

The serum biochemical characteristics after 4 weeks of dietary treatment are shown in Table 5. The serum glucose, FFA, triglyceride, and acetoacetic acid concentrations were not significantly different among the three diet groups, but β-hydroxybutyric acid concentrations were significantly higher in the fish oil-fed rats than in the lard-fed rats, suggesting an increase in β-oxidation in the livers of fish oil-fed rats. 

**Table 5:** Concentration of serum energy substrates in rats fed each diet.

	Soybean oil			Fish oil			Lard		
Glucose, mg/dl	196.3	±	22.4	167.3	±	13.7	151.7	±	10.8
FFA, mEq/l	0.176	±	0.029	0.204	±	0.039	0.118	±	0.025
Triglyceride, mg/dl	189.9	±	34.6	204.5	±	38.7	146.2	±	25.3
Acetoacetic acid, µM	3.611	±	1.221	4.264	±	1.768	2.864	±	1.418
β-hydroxybutyric acid, µM	13.483	±	3.875^ab^	19.135	±	5.057^a^	4.313	±	1.395^b^

Values are means ± SE for 6 rats. Different superscripts indicate there is a significant difference between two groups (p < 0.05).

FFA; free fatty acids.

### MyHC isoform composition and transcripts in EDL and soleus muscle

**Figure 1:**
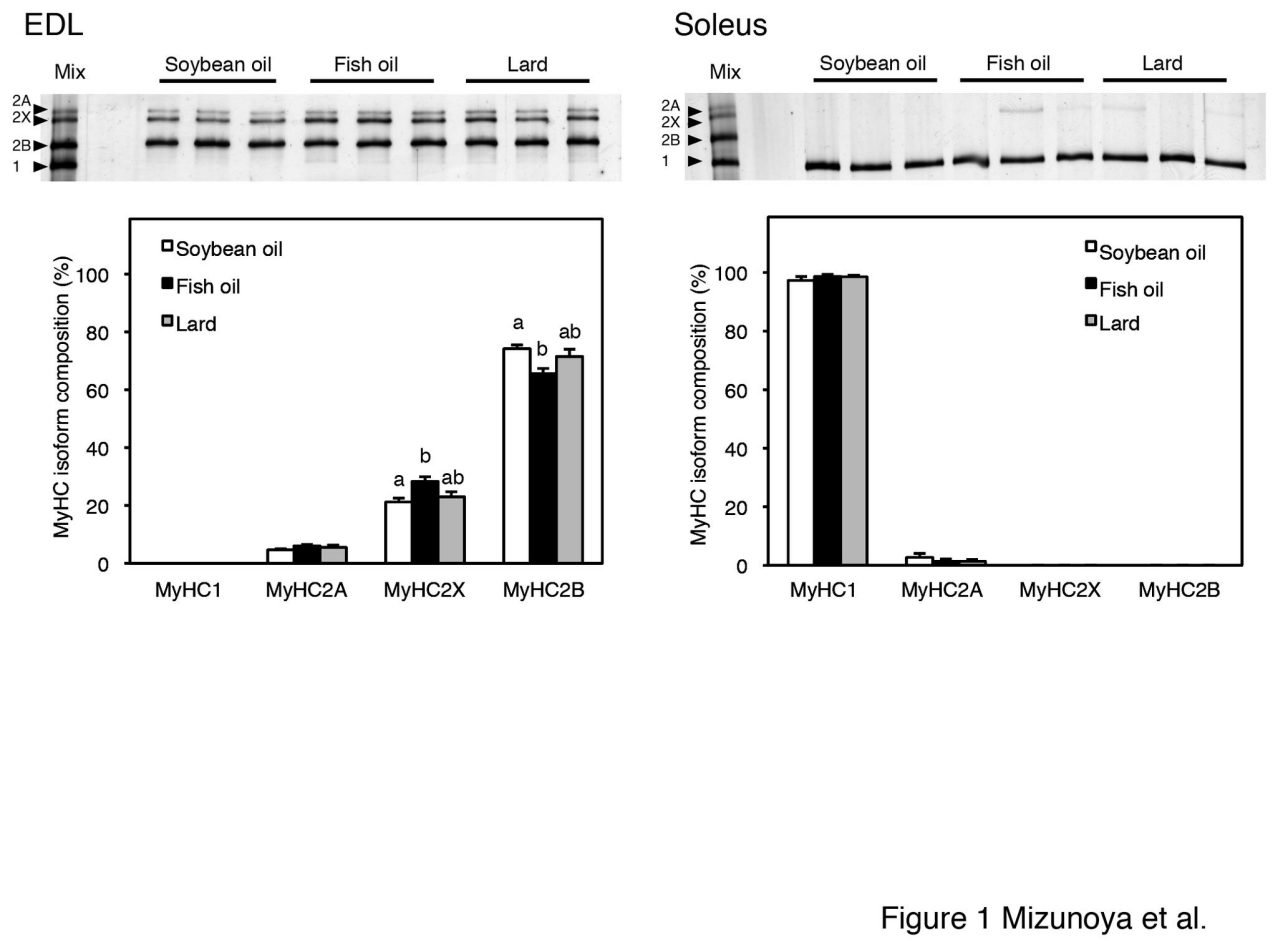
Relative protein contents of myosin heavy chain isoforms (MyHC1, 2A, 2X, and 2B) in EDL and soleus muscles from soybean oil-fed (open bars), fish oil-fed (filled bars), and lard-fed (gray bars) rats. The values are means ± SE for six rats. The bands above the graphs represent the MyHC isoforms detected in silver-stained, high-resolution, 8% SDS-PAGE gels for three representative rats from each group. Relative MyHC2X and 2B isoform contents in the EDL differed significantly (p < 0.05) between the soybean oil-fed and fish oil-fed rats. In the soleus, no significant differences were observed in MyHC isoform expression. A mix of rat EDL and soleus samples was used as the four MyHC isoform references (left lane). Different superscripts indicate a significant difference between two groups (p < 0.05, one-way ANOVA; post hoc: Tukey–Kramer multiple-comparison test).

MyHC isoform composition is a common index for determining overall skeletal muscle fiber type. Figure 1 illustrates the MyHC electrophoresis pattern in the EDL and soleus of rats from each dietary treatment and their densitometrically analyzed results, which indicate MyHC isoform protein composition. The composition of MyHC isoforms was obviously different between the EDL and soleus. The EDL was composed of mainly MyHC2B, 2X, and 2A, whereas the soleus was composed exclusively of MyHC1. The mean percentage of MyHC2X in the EDL of fish oil-fed rats was significantly higher than that in the EDL of soybean oil-fed rats (27.2 ± 2.1 vs. 21.2 ± 1.3%, p < 0.05), while MyHC2B was significantly lower (65.6 ± 1.7 vs. 74.2 ± 1.3%, p < 0.05). In contrast to the EDL, there was no significant difference in the MyHC composition of the soleus among the dietary treatments. In contrast to the tissue weights and serum metabolites, the MyHC isoform composition values of the lard-fed rats were intermediate among the three treatment groups.

**Figure 2:**
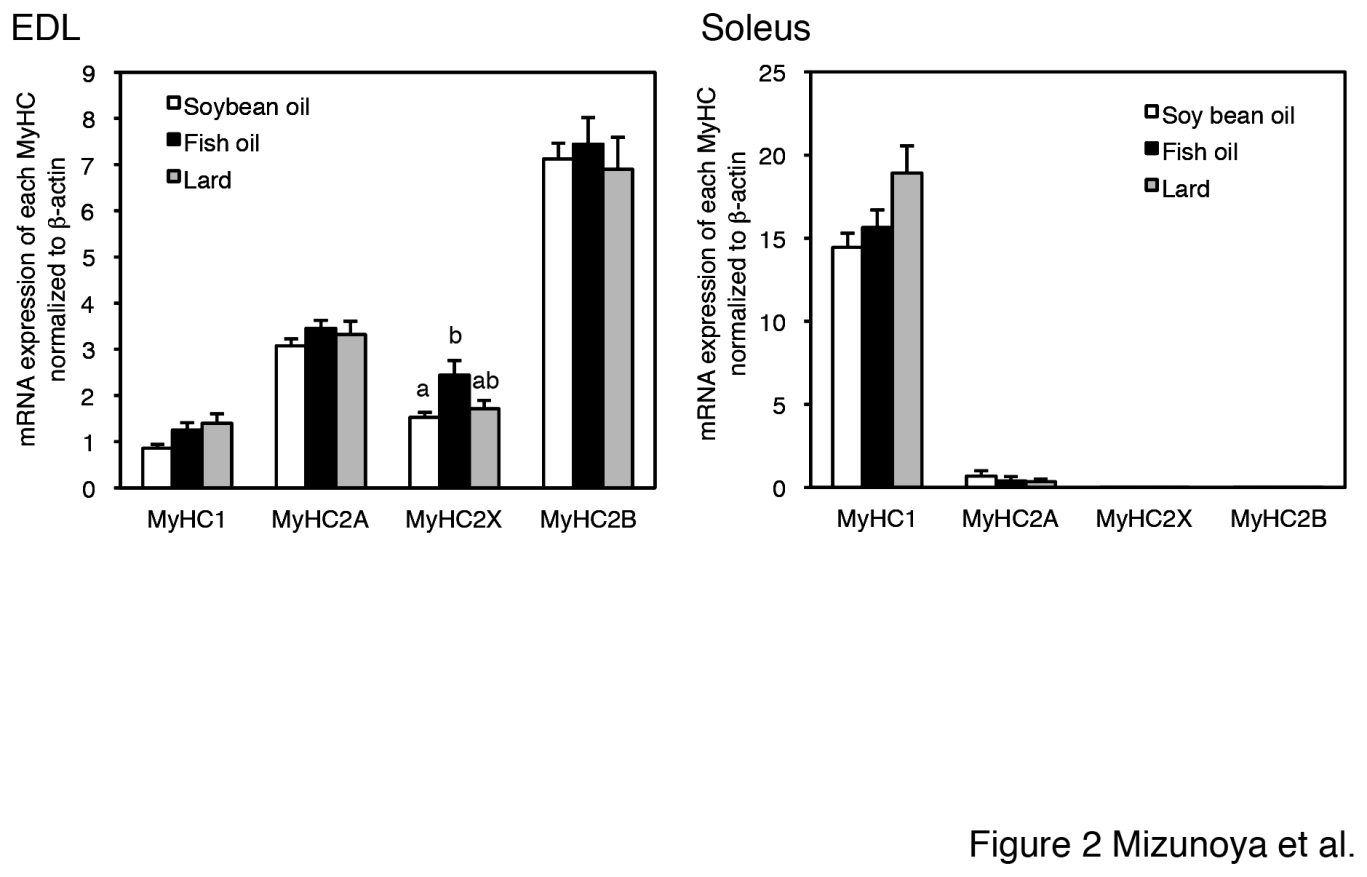
Relative myosin heavy chain isoform (MyHC1, 2A, 2X, and 2B) mRNA expression in EDL and soleus muscles from soybean oil-fed (open bars), fish oil-fed (filled bars), and lard-fed (gray bars) rats. Values are calculated based on the ΔCt method (where Ct is the threshold cycle) [25], using the following formula: 2^-(Ct(target gene) – Ct(β-actin))^, which gives the relative target gene expression compared to β-actin expression. The values are means ± SE for six rats. Relative MyHC2X mRNA expression levels in the EDL differed significantly (p < 0.05) between soybean oil-fed and fish oil-fed rats. Different superscripts indicate a significant difference between two groups (p < 0.05, one-way ANOVA; post hoc: Tukey–Kramer multiple-comparison test).

The mRNA levels of the respective MyHC isoforms normalized by β-actin largely corresponded to the protein composition patterns of the EDL and soleus, that is, MyHC2A, 2X, and 2B were higher in the EDL, and MyHC1 was higher in the soleus (Figure 2). Similar to the protein results, MyHC2X mRNA expression was significantly higher in fish oil-fed rats than in soybean oil-fed rats.

### Transcript and protein levels of genes involved in metabolism, mitochondria, and transcription factors in EDL and soleus muscle

**Figure 3:**
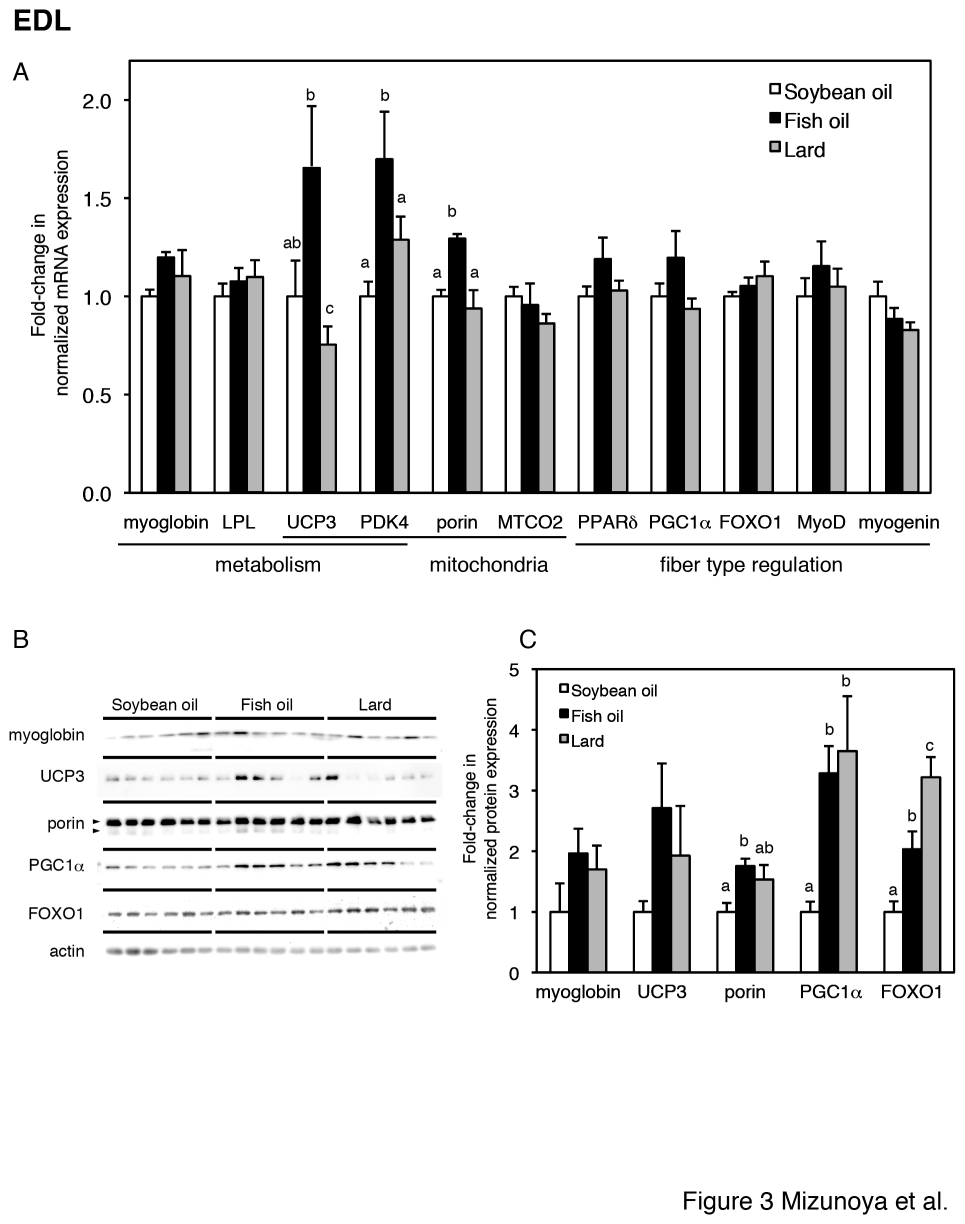
Gene and protein expression in the EDL muscle (A–C). (A) Relative expression levels of genes for energy metabolism (myoglobin, LPL, UCP3, PDK4), mitochondrial proteins (porin, MTCO2, UCP3 and PDK4), and transcription factors (PPARδ, FOXO1, MyoD, myogenin) and a coactivator (PGC1α) for muscle fiber type regulation in EDL muscles from soybean oil-fed (open bars), fish oil-fed (filled bars), and lard-fed (gray bars) rats. Each gene was normalized to β-actin mRNA. Values are expressed as fold changes compared with the soybean oil-fed group. (B) Protein expression levels of fiber type-related markers (myoglobin, UCP3, porin, PGC1α, and FOXO1) and loading control (actin) in the EDL, with densitometry quantification in panel C. The two arrowheads in porin indicate two porin species at 32 and 36 kDa [51], which were summed in panel C. The values are means ± SE for six rats. Different superscripts indicate a significant difference between two groups (p < 0.05, one-way ANOVA; post hoc: Tukey–Kramer multiple-comparison test).

**Figure 4:**
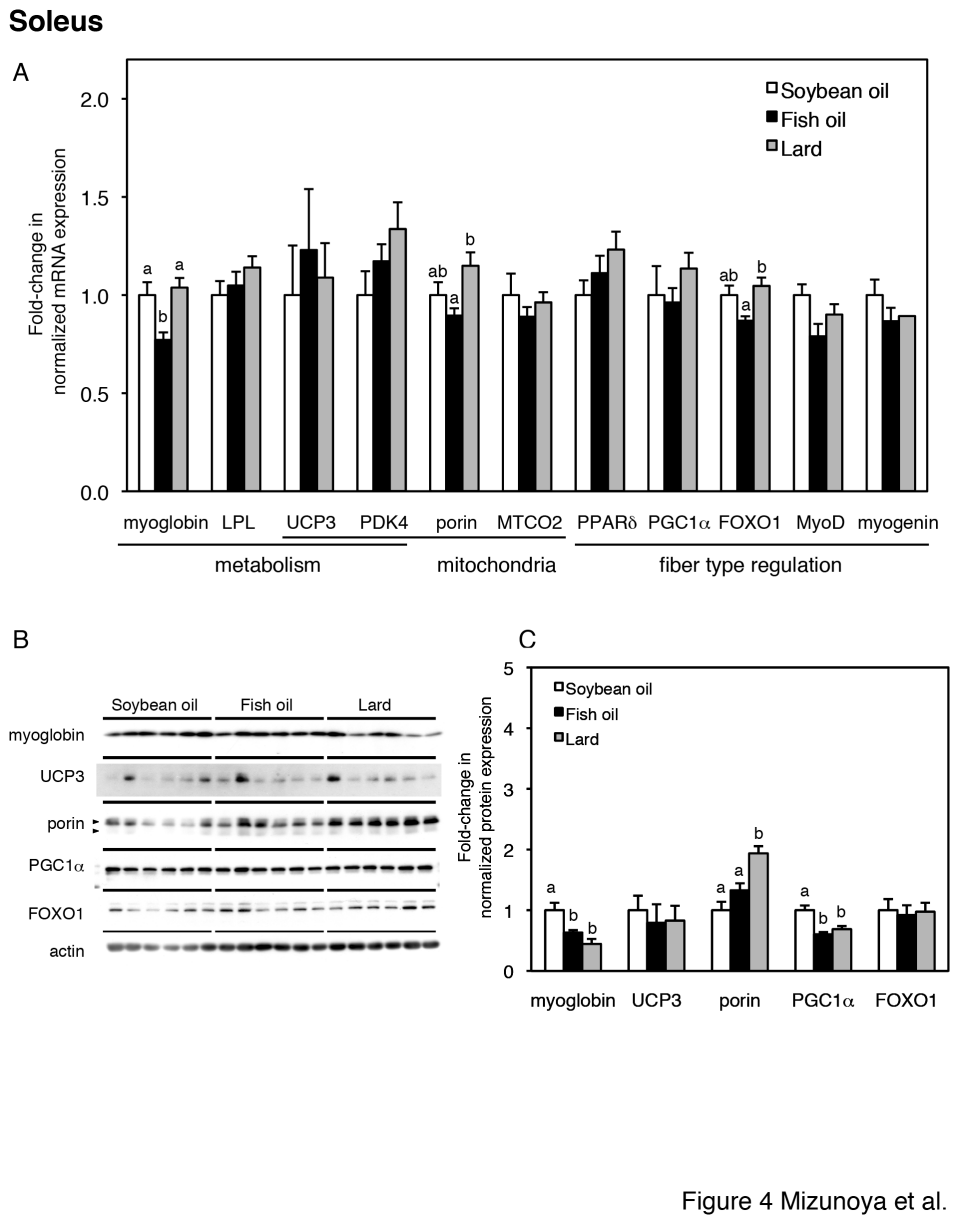
Gene and protein expression in the soleus muscle (A–C). (A) Relative expression levels of genes for energy metabolism (myoglobin, LPL, UCP3, PDK4), mitochondrial proteins (porin, MTCO2, UCP3 and PDK4), and transcription factors (PPARδ, FOXO1, MyoD, myogenin) and a coactivator (PGC1α) for muscle fiber type regulation in soleus muscles from soybean oil-fed (open bars), fish oil-fed (filled bars), and lard-fed (gray bars) rats. Each gene was normalized to β-actin mRNA. Values are expressed as fold changes compared with the soybean oil-fed group. (B) Protein expression levels of fiber type-related markers (myoglobin, UCP3, porin, PGC1α, and FOXO1) and loading control (actin) in the soleus, with densitometry quantification in panel C. The two arrowheads in porin indicate two porin species at 32 and 36 kDa [51], which were summed in panel C. The values are means ± SE for six rats. Different superscripts indicate a significant difference between two groups (p < 0.05, one-way ANOVA; post hoc: Tukey–Kramer multiple-comparison test).

To further reveal the effect of the three dietary fats on skeletal muscle fiber type, transcript levels of genes related to (i) energy metabolism (myoglobin, LPL, UCP3, and PDK4); (ii) mitochondrial protein (porin, MTCO2, UCP3, and PDK4); and (iii) transcription factors (PPARδ, FOXO1, MyoD and myogenin) and a coactivator (PGC1α) for muscle fiber type regulation were analyzed by quantitative RT-PCR. The expression of the transcripts of EDL and soleus is shown in Figure 3A and 4A respectively. In EDL muscle of fish oil-fed rats, the transcript levels of PDK4 and porin were significantly increased compared to soybean oil-fed and lard-fed rats, and the transcript level of UCP3 was significantly higher than that in lard-fed rats but not significantly different than that in soybean oil-fed rats (Figure 3A). The dietary fat type did not alter the mRNA expression level of several transcription factors of muscle fiber type regulators in the EDL muscle .

The protein expression of myoglobin, UCP3, porin, PGC1α, and FOXO1 in the EDL muscle was examined (Fig. 3BC). In fish oil-fed rats, the protein level of porin was significantly higher than that in soybean oil-fed rats, which corresponded to the transcript difference. In contrast to the transcript results, in fish oil-fed and lard-fed rats, the protein levels of PGC1α and FOXO1 were significantly higher than those in soybean oil-fed rats, and the level of FOXO1 in lard-fed rats was significantly higher than that in fish oil-fed rats. 

In soleus muscle of fish oil-fed rats, the transcript levels of myoglobin was significantly decreased compared to soybean oil-fed and lard-fed rats, and the transcript levels of porin and FOXO1 were significantly lower than that in lard-fed rats but not significantly different than that in soybean oil-fed rats (Figure 4A). The dietary fat type did not alter the mRNA expression level of transcription factors of muscle fiber type regulators except for FOXO1 in the soleus muscle.

The protein expression of myoglobin, UCP3, porin, PGC1α and FOXO1 in the soleus muscle was also examined (Fig. 4BC). The level of myoglobin in fish oil-fed rats was significantly lower than that in soybean oil-fed rats, which corresponded to the transcript difference. However, the level of myoglobin in lard-fed rats was also significantly lower than that in soybean oil-fed rats, though there was no significant difference in the transcript level. In lard-fed rats, the protein level of porin was significantly higher than that in soybean oil-fed and fish oil-fed rats, which was similar to the transcript difference. In contrast to the transcript results, in fish oil-fed and lard-fed rats, the protein levels of PGC1α was significantly lower than those in soybean oil-fed rats. 

### Composition of fatty acids in serum and muscle

**Figure 5:**
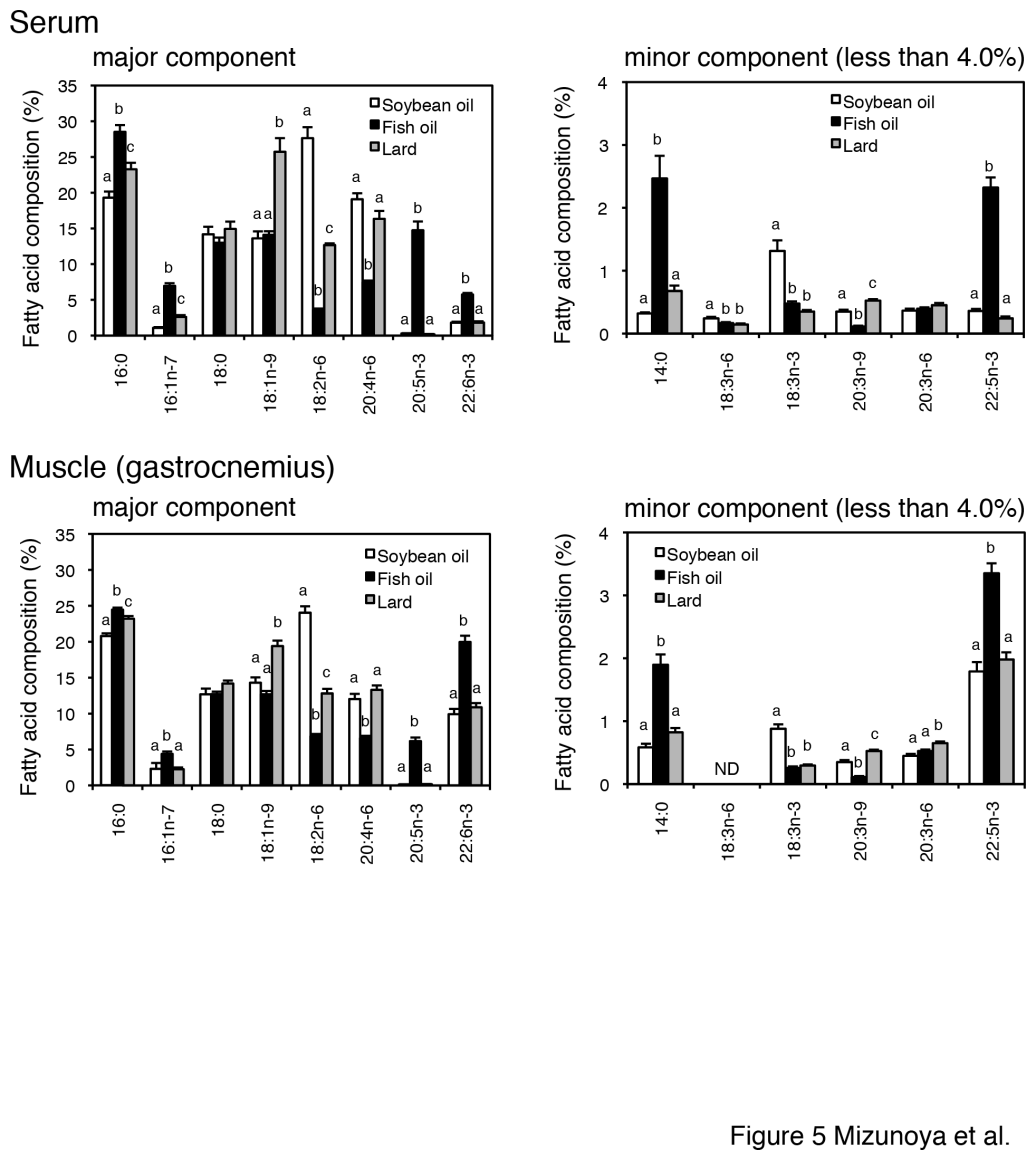
Comparison of the distribution of fatty acids in the serum and gastrocnemius muscle (A–D). (A) Major fatty acids (more than 4.0%) and (B) minor fatty acids (less than 4.0%) composing lipids in sera from soybean oil-fed (open bars), fish oil-fed (filled bars), and lard-fed (gray bars) rats. (C) Major fatty acids (more than 4.0%) and (D) minor fatty acids (less than 4.0%) composing lipids in gastrocnemius muscles from soybean oil-fed (open bars), fish oil-fed (filled bars), and lard-fed (gray bars) rats. The values are means ± SE for six rats. Different superscripts indicate a significant difference between two groups (p < 0.05, one-way ANOVA; post hoc: Tukey–Kramer multiple-comparison test). 14:0, myristic acid; 16:0, palmitic acid; 16:1n-7, palmitoleic acid; 18:0, stearic acid; 18:1n-9, oleic acid; 18:2n-6, linoleic acid; 18:3n-6, γ-linolenic acid; 18:3n-3, α-linolenic acid; 20:3n-9, mead acid; 20:3n-6, dihomo-γ-linolenic acid; 20:4n-6, arachidonic acid; 20:5n-3, eicosapentaenoic acid (EPA); 22:5n-3, docosapentaenoic acid; 22:6n-3, docosahexaenoic acid (DHA); ND, not detected.

To assess the delivery of fatty acids derived from the respective dietary fats to skeletal muscles, we examined the fatty acid composition of the total lipids in the serum and gastrocnemius (Figure 5). Significantly higher relative amounts of n-3 PUFA (EPA, DHA, and docosapentaenoic acid) were observed in both the sera and muscle of fish oil-fed rats. Furthermore, diminished amounts of linoleic acid (n-6 PUFA) and monounsaturated fatty acids, as well as reduced n-6/n-3 fatty acid ratios, were observed in the fish oil-fed rats. The n-6/n-3 ratios of lipids in muscles were 2.94 ± 0.22, 0.47 ± 0.01, and 2.03 ± 0.11 for the soybean oil-fed, fish oil-fed, and lard-fed groups, respectively. Significant differences were observed between the soybean oil-fed and fish oil-fed groups, between the fish oil-fed and lard-fed groups, and between the soybean oil-fed and lard-fed groups (p < 0.01 by ANOVA and Tukey-Kramer test).

## Discussion

The present study was designed to examine the effects of different dietary fats derived from plants, animals, and fish—which show rather dissimilar fatty acid profiles—on the skeletal muscle fiber types. The contractile as well as metabolic properties of the skeletal muscle was analyzed with reference to the MyHC isoform composition—which is the most common protein marker used to delineate muscle fiber types—and that of the mRNA and protein levels of metabolism-related genes, in both slow-type dominant (soleus) and fast-type dominant (EDL) muscle tissues. Our study showed that the EDL muscle had a significantly higher MyHC2X and lower MyHC2B composition in the fish oil-fed rats compared to soybean oil-fed rats. The MyHC isoform composition of the lard-fed rats was intermediate between that of the fish oil-fed and soybean oil-fed rats. This alteration in the composition of the MyHC isoforms was accompanied by a significantly higher expression of MyHC2X mRNA in the fish oil-fed rats compared to soybean oil-fed rats. In agreement with this alteration of contractile genes, the mRNA and protein expression of some oxidative metabolism-related genes was increased in the fish oil-fed rats compared to soybean oil-fed rats. However, the effect of dietary fat on MyHC composition and expression of oxidative metabolism-related genes, in the soleus muscle was not in agreement with that observed in the EDL. Thus, our study indicates a dietary fat-/fatty acid-dependent, differential regulation of contractile and metabolic properties between fast-type and slow-type muscles.

Many aspects of muscle metabolism are believed to correspond to MyHC isoform composition. The biological significance between type 2X and type 2B fibers has not been fully elucidated. However, a previous study reported that the activity of succinate dehydrogenase, a metabolic enzyme involved in the citric acid cycle, was higher in the intermediate type 2X fibers than in the fastest type 2B fibers [6]. In the present study, the increase in type 2X was concomitant with upregulation of oxidative metabolism-related factors in fish oil-fed rats. Overall, in the EDL muscle, fish oil intake induced higher expression of oxidative metabolism-related factors than soybean oil or lard intake, which are usually expressed at higher level in slow- or intermediate-type fibers. Significant increases in the mRNA expression of mitochondrial UCP3, PDK4, and porin were observed in the fish oil-fed rats compared with soybean oil or lard-fed rats. In other *in vitro* studies, similar results were observed, i.e. n-3 PUFA supplementation upregulated the expression of PDK4 [27] and UCP3 [28] in differentiated C2C12 myotubes. PDK4 expression in the skeletal muscle of mice fed n-3 PUFA concentrate has been reported [29]. Male C57BL/6N mice were fed a high-fat diet containing 35% (w/w) fat (mainly corn oil) or a diet supplemented with n-3 PUFA concentrate (46% DHA, 14% EPA), which replaced 15% (w/w) of dietary fats for eight weeks. In the study, gastrocnemius muscle PDK4 expression was not affected by the dietary intake of n-3 PUFA. These results are contrary to those of the present study, which revealed significant PDK4 upregulation with fish oil intake (Figure 3A). At present, the causes of disagreement are unclear. However, it may be due to some differences in experimental conditions such as the animals, dietary fat levels, n-3 PUFA amounts, and experimental periods. It is also reported that DHA and EPA have different biological activities on lipid metabolism [18]. The fish oil used in our experiment contained a much higher EPA (20%) content than DHA content (8%). Thus, EPA, rather than DHA, may be primarily responsible for promoting PDK4 upregulation.

One possible biological pathway for the alterations of the expression of contractile and metabolic genes induced by dietary fat in the EDL muscle is the modulation of transcription factors such as PPARδ. PPARs are ligand-dependent nuclear receptors that belong to the superfamily of nuclear transcription factors. PPARδ is believed to be the key nuclear receptor for regulating muscle fiber type. Over-expression of constitutively active, muscle-specific VP16-PPARδ was previously reported to enhance mitochondrial gene expression in association with a fiber type switch from fast glycolytic type 2 fibers to slow oxidative type 1 fibers [3]. Fatty acids are ubiquitous biological molecules that are used as metabolic fuels, covalent regulators of signaling molecules, and essential components of cellular membranes. Fatty acids are natural, endogenous ligands for PPARs, and they mediate adaptive metabolic responses to changes in systemic fuel availability [30–34]. Interestingly, PPAR affinity varies depending on the fatty acid species. For example, EPA is a much more potent activator of PPARα in primary hepatocytes than arachidonic acid, an n-6 PUFA [35]. Using luciferase reporter assays, Forman et al. demonstrated that PUFAs such as linoleic acid, arachidonic acid, and EPA are much more potent activators of PPARδ at a concentration of 30 µM [31]; this level is physiologically relevant because mammalian serum free fatty acid concentrations can be greater than 100 µM. Therefore, it is possible that EPA, which is abundant in the fish oil, could function as a PPARδ agonist and affect the expression of contractile and metabolic genes in the skeletal muscles. However, it remains to be elucidated whether the PPARδ functions are different between the EDL and the soleus.

A difference in drug reactivity between fast-type and slow-type muscles has also been observed in PPARδ agonist (GW501516)-treated animals [36]. In that report, the authors described that in all their gene expression studies, the maximal effects of PPARδ activation were predominantly detected in fast-twitch (quadriceps and gastrocnemius) and not slow-twitch (soleus) muscles. Cresser et al. showed that glycolytic fibers (white gastrocnemius) increased their capacity for oxidative metabolism more robustly than oxidative fibers (soleus and red gastrocnemius) in response to treatment with GW501516 [37]. In our results, the EDL showed a greater response to alterations of dietary fat in contractile and metabolic factors compared to the soleus muscle. Thus, it seems likely that activation or function of PPARδ induced by fatty acids derived from dietary fat is more prominent in fast-type muscles such as EDL.

Fatty acids are transported throughout the body in esterified and nonesterified forms. Since fatty acids are bioactive substances, it is important to note that fatty acids from the diet could reach the target tissues. Indeed, the fatty acid composition of the muscles was reflected in part by the fatty acid composition of the diets, with high levels of EPA and DHA in the muscle of rats fed with fish oil diet, high levels of linoleic acid in the muscle of rats fed with soybean oil diet, and high levels of oleic acid in the muscle of rats fed with lard diet (Figure 5). Consistent with this observation, previous studies have reported that the fatty acid composition of muscle lipids is influenced by the type of dietary fat in the diet [38,39]. In one of these studies, the overall effects of the dietary fatty acids were similar in both soleus and EDL muscles [38].

 PGC1α is a transcriptional coactivator that was first discovered by Spiegelman’s group [40] as a cold-inducible protein that binds to PPARγ and increases the expression of UCP1 in the brown fat of mice. Forced expression of PGC1α in the skeletal muscle of transgenic mice triggers the formation of type 1 oxidative muscle fibers [41]. In our experiments, there was a greater increase in the PGC1α protein in the EDL muscle in fish oil-fed and lard-fed rats than in soybean oil-fed rats. Although the slow MyHC1 composition did not increase in the EDL muscle, the increase of intermediate type 2X occurred in our experiments. 

The level of FOXO1 protein was significantly increased in the fish oil-fed and lard-fed rats compared with soybean oil-fed rats. Recent evidence supports the involvement of the transcription factor FOXO1 in regulating the adaptive metabolism of muscle. FOXO1 recruits the fatty acid translocase CD36 to the plasma membrane, leading to increased fatty acid uptake and oxidation in C2C12 myogenic cells [42]. The reciprocal role of FOXO1 in muscle fiber type regulation must be noted here. Transgenic mice specifically over-expressing FOXO1 in skeletal muscle exhibit decreased expression of many genes related to the structural proteins of type 1 slow fibers [43]. By contrast, conditional ablation of FOXO1 expression in the soleus muscle leads to reduced slow fiber formation and increased fast fiber formation, a phenotype similar to that observed upon FOXO1 over-expression [44]. The mechanism through which FOXO1 plays a critical role in muscle fiber type regulation remains controversial.

Protein levels of PGC1α and FOXO1 in EDL were increased in fish oil-fed and lard-fed rats compared to soybean oil-fed rats, however mRNA levels were not significantly upregulated. It is likely that these two proteins were post-translationally modified in response to dietary fat, though this was not directly assessed. Until now, cytokines and phosphorylation have been implicated in the control of PGC1α degradation rates [45]. It has also been reported that acetylation is involved in the post-translational control of PGC1α protein levels [46]. Additionally, it has been suggested that insulin signaling leads to FOXO1 polyubiquitination, resulting in downregulation via proteasome-dependent degradation [47]. FOXO1 is phosphorylated by protein kinase B (PKB) in the insulin signaling pathway and consequently excluded from the nucleus. Phosphorylated FOXO1 is then ubiquitinated in the cytoplasm, followed by degradation through the 26S proteasome. Thus, if dietary fat/fatty acids affect signaling enzymes such as kinases, phosphorylases, acetylases, and deacetylases, protein levels of PGC1α or FOXO1 could be altered independently from mRNA levels.

Surprisingly, although lard containing the abundant saturated fatty acids was expected to show the lowest oxidative phenotypes, the lard-fed rats showed intermediate phenotypes between fish oil- and soybean oil-fed rats in many results of the present study. This might be due to that high oleic acid content in lard. The 25-year follow-up study showed that the incidence of cardiovascular disease in middle-aged men from the Mediterranean area of Southern Europe was relatively lower than that expected in men with similar cholesterol concentration [48]. The traditional Mediterranean diet is characterized by a high consumption of olive oil, which is rich in monounsaturated fatty acids, mainly oleic acid. The low incidence of cardiovascular disease observed in these populations could be related to the beneficial effects of diets rich in oleic acid. In agreement with this notion, it is reported that the intake of oleic acid-rich diet improved the abnormal distribution of LDL and HDL in mildly obese normolipidemic women [49].

It is important to assume pre-feeding muscle fiber type prior to dietary intervention. Before the dietary intervention, rats were fed a commercial diet (CRF-1), where the fatty acid composition was similar to the soybean oil diet (approximately 50% linoleic acid and 24% oleic acid). Thus, initial conditions may have been similar to the soybean oil-fed group. Previously, we reported the effects of 48-h food deprivation on muscle fiber type properties [50].. In that study, rats of the same strain, age, breeder, and commercial diet (CRF-1) were used and MyHC composition of the control group was very similar to this experiment in both EDL and soleus muscle. Thus, it is assumed that the muscle fiber type was largely unaltered during the 4-week experimental period in this dietary fat intervention experiment.

In conclusion, the intake of different types of dietary fat showed an evident effect on rat skeletal muscle contractile and metabolic gene expressions in the EDL, a fast-type dominant muscle tissue. In particular, fish oil intake showed more oxidative characteristics than soybean oil intake. In contrast, the effect of dietary fat type on the soleus, a slow-type dominant muscle tissue, was less evident. Further studies are required to clarify the effects of different types of dietary fats on various muscle functions, and to unravel the mechanism underlying the action of slow-type and fast-type muscles.
